# Active food packaging films from alginate and date palm pit extract: Physicochemical properties, antioxidant capacity, and stability

**DOI:** 10.1002/fsn3.3093

**Published:** 2022-10-06

**Authors:** Khaoula Khwaldia, Yassine M'Rabet, Abdennacer Boulila

**Affiliations:** ^1^ Laboratoire des Substances Naturelles Institut National de Recherche et d'Analyse Physico‐chimique (INRAP) Sidi Thabet Tunisia

**Keywords:** antioxidant packaging, biopolymers, date palm by‐products, date seeds, edible films, phenolic compounds

## Abstract

Date palm pits are highly available and inexpensive palm date by‐products, representing a valuable source of natural antioxidants, particularly phenolic compounds. Date palm pit extract (DPPE) was prepared from these waste products and characterized for its phenolic content and in vitro antioxidant activity. Profiling DPPE by liquid chromatography coupled with mass spectrometry (LC/MS) showed the presence of dimers and trimers of (epi)catechin as the main constituents. Alginate‐based films with four increasing concentrations of DPPE (10%, 20%, 30%, and 40% w/w) were prepared by the casting method. DPPE incorporation reduced solubility values of alginate films by 37%–64% and their surface wettability by 72%–111%. The incorporation of 10% DPPE improved water vapor barrier properties and increased tensile strength (TS) and elongation at break (%E) of alginate films by more than 23%, 50%, and 45%, respectively. The film containing 40% DPPE showed the lowest loss of phenolic content (32%), DPPH (1,1‐diphenyl‐2‐picrylhydrazyl) scavenging activity (38%), and ferric reducing antioxidant power (FRAP) (30%) after storage for 3 months.

## Graphical abstract

Date palm pit extract (DPPE), characterized for its phenolic compounds' composition and antioxidant activity, displayed a high antioxidant potential. Alginate–DPPE active films showed enhanced water barrier, mechanical, and antioxidant properties. These films are able to retard lipid oxidation in foods during storage.

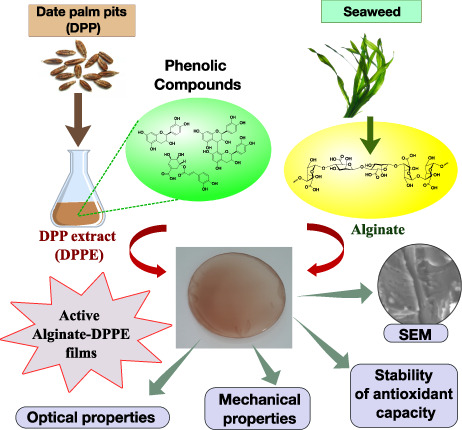

## 1 INTRODUCTION

The urgent need to seek alternatives to conventional plastic‐based packaging materials has been mainly motivated by the environmental and social costs related to their fabrication, use, disposal, and treatment as well as the increased consumer demand for sustainable, recyclable, and environmentally friendly packaging materials. The latest COVID‐19 pandemic showed the limits of the traditional waste recycling system, especially accented by takeaway sales and home delivery, imposed by confinement measures, which led to a growing demand for single‐use plastic packaging (Barone et al., 2021).

Agro‐industrial waste and by‐products have been promoted in many countries as sustainable alternatives to conventional raw materials for manufacturing and green‐energy production. The development of biodegradable films from fruit and vegetable by‐products constitutes an excellent strategy to reuse these residues taking advantage of their bioactive compounds, limiting their disposal problems, and contributing to the circular economy model (Paidari et al., 2021).

Bio‐based polymers including polysaccharides, proteins, and lipids have been used to develop edible and biodegradable packaging materials which showed their efficacy in maintaining quality and prolonging the shelf life of many food products (Aloui et al., 2014; Nicolau‐Lapeña et al., 2021; Paidari et al., 2021). Alginate, one of the most popular marine polysaccharides, has widely served as an interesting polymeric supporting matrix for packaging materials due to its high abundance, low cost, nontoxicity, biocompatibility, nonimmunogenicity, biodegradability, stability, and emulsifying and film‐forming properties (Arroyo et al., 2020; Parreidt et al., 2018; Wang et al., 2019). Sodium alginate‐based films exhibited good oxygen and grease barrier properties as well as a glossy appearance and were water‐soluble, tasteless, and odorless (Gheorghita et al., 2020). Alginate‐based coatings were successfully applied to many food products, including fruits and vegetables (Aloui et al., 2021; Chen et al., 2021), meat (Konuk Takma & Korel, 2018), seafood (Nie et al., 2018), and cheese (Lucera et al., 2014), to improve their quality and prolong their shelf life. However, due to their limited mechanical property, water barrier, and thermal stability, many strategies have been adopted to improve the functional properties of sodium alginate‐based packaging materials, such as blending with other polymers (Zhang et al., 2021b), cross‐linking (Priya et al., 2014), incorporation of nano‐reinforcing agents (Arroyo et al., 2020), and addition of active compounds (Aloui et al., 2014; Nie et al., 2018).

Ensuring higher stability and gradual release of active agents and maintaining critical loads for a prolonged storage time, alginate films and coatings had served as effective vehicles for antimicrobial and antioxidant agents to enhance food quality and shelf life (Parreidt et al., 2018). Several researchers have recently developed active alginate‐based matrices incorporating natural antioxidants, especially polyphenols, and demonstrated their efficacy in reducing oxidation by preventing free radical generation and scavenging reactive oxygen species (Biao et al., 2019; Moreno et al., 2020; Ruan et al., 2019). Being nonvolatile compounds, polyphenols represent a good alternative for essential oils which could negatively affect food sensory attributes or may be lost by volatilization throughout storage (Vital et al., 2018; Zhang et al., 2021a). Recently, recovering phenolic compounds from cheap raw materials such as wastes and by‐products from food processing industries has emerged as a promising approach to minimize the harmful impacts of waste disposal and provide renewable high‐added value products (Andrade et al., 2019; Dilucia et al., 2020).

Being the earliest tree crop based on archaeobotanical data and one of the most consumed fruit crops in many arid and semiarid regions of the Middle East and North Africa (Sarah et al., 2022), date palm has been widely studied owing to its nutritional, functional, and health properties (Maqsood et al., 2020; Otify et al., 2019). However, there is relatively limited research and patents on reusing date palm by‐products (Farag et al., 2021). Among the main palm date by‐products, date pits, which are produced in large quantities reaching in some countries 30% of the harvested date fruits, are mainly used as a soil amendment or animal feed (Oladzad et al., 2021). Date pits are known as a valuable source of nutrients and natural antioxidants including vitamins, phenolic compounds, tocopherols, and carotenoids (Farag et al., 2021). Accordingly, some studies have reported their use as a coffee substitute or for the extraction of date palm oil for cosmetic and pharmaceutical applications.

Recently, date pits raw products have shown good efficiency as edible‐coating additives. Ahmed et al. (2020) showed that the incorporation of date pit oil at 2% in wax‐coating formulations significantly improved guava fruit shelf life. Low‐cost biodegradable films developed from corn starch and date pit powder revealed enhanced mechanical, antioxidant, and insulating properties (Alqahtani et al., 2021). In this context, date seeds rich in active compounds represent a suitable candidate for producing biodegradable films and coatings with increased functionality and environmental sustainability. Therefore, this study aimed at evaluating the effect of date palm pit extract (DPPE) on the physicochemical properties, antioxidant capacity, and stability of alginate films.

## 2 MATERIALS AND METHODS

### 2.1 Materials and reagents

In December 2019, pits from the fruits of date palm (*Phoenix dactylifera* L., Variety Deglet Nour) were kindly provided by a local date palm processing industry in Tozeur, a large oasis located in southwest Tunisia (Latitude of 33°55′ 10.85′′ N; Longitude of 8° 08′ 0.67′′ E). After being thoroughly washed with distilled water, palm date pits were oven‐dried at 45°C for 48 h, finely ground with a Kinematica Polymix PX‐MFC 90 D mill, and then stored at +4°C in amber glass jars until extraction. All solvents and reagents used in this study, sodium alginate with an average molecular weight of 80,000 Da, and glycerol as a plasticizer were purchased from Sigma‐Aldrich.

### 2.2 Preparation of date palm pit extract

Date palm pit extract was prepared by reflux extraction as previously described by Souissi et al. (2018) with some modifications. Briefly, 1.25 g of date pits powder was extracted under reflux with a 100 ml water–ethanol mixture (90:10) for 1 h. The obtained hydro‐ethanolic extracts were filtered through a P1 glass frit (porosity 100–160 μm) and the solvent was removed under reduced pressure using an IKA rotary evaporator. Finally, DPPE was freeze‐dried (Christ‐Alpha) and stored in amber glass bottles at −20°C until use.

### 2.3 Chemical characterization of DPPE

#### 2.3.1 Determination of condensed tannin content

The DPPE condensed tannin content (CTC) was carried out as described by Aires et al. (2016). After each extraction, an aliquot of 50 ml was mixed with 5 ml HCl (37%) and 10 ml formaldehyde. The mixture was left under reflux for 30 min and then filtered and washed with distilled water. The residue was placed in a drying oven at a temperature of 105 ± 3°C until the mass stabilization (*MS*). Stiasny's index (SI) relative to the dry mass of the extract (*DW*) was calculated according to the following equation:

$$\mathrm{SI}\left(\%\right)=\frac{\mathrm{MS}}{\mathrm{DW}}\times 100$$

#### 2.3.2 Determination of total polyphenolic content

The total phenolic content (TPC) of DPPE was determined by the Folin–Ciocalteu (F‐C) method (Singleton & Rossi, 1965). An aliquot of DPPE (100 μl) was added to 1 ml of freshly diluted 10‐fold F‐C reagent. After 5 min, 2.5 ml of Na_2_CO_3_ (75 g/L) was added followed by 1 h incubation in dark at room temperature. The absorbance was measured at 760 nm (JASCO V‐630). Gallic acid was used as a calibration standard and TPC was expressed as mg gallic acid equivalents per gram of freeze‐dried extract (mg GAE/g).

#### 2.3.3 Determination of total anthocyanin content

The determination of total anthocyanin content (TAC) was assessed by the pH‐differential method based on the absorbance measurements of diluted extracts with buffer solutions at pH 1.0 and 4.5, at 520 and 700 nm (Giusti & Wrolstad, 2001). Briefly, extracts were diluted separately with 0.025 M hydrochloric acid–potassium chloride buffer (pH = 1) and 0.4 M sodium acetate buffer (pH = 4.5) until the absorbance was within a linear range. The absorbance of each solution was measured at 520 and 700 nm. The total anthocyanin content (TAC) expressed as milligrams (mg) of cyanidin‐3‐glucoside equivalent/g dry weight of extract (mg CGE/g DW) was calculated following the equation:

$$\mathrm{TA}=\frac{\left(A\times \mathrm{MW}\times \mathrm{DF}\times 10^{3}\right)}{\varepsilon \times l}$$

where *A* the absorbance is (A₅₂₀–A₇₀₀)_pH 1.0_ – (A₅₂₀–A₇₀₀)_pH 4.5_; *MW*: molecular weight of cyanidin‐3‐glucoside (449,38 g/mol); *DF*: dilution factor; *l*: the cell path length (1 cm); *ɛ*: the coefficient of molecular extinction = 26.900 L mol^−1^ cm^−1^ (Giusti & Wrolstad, 2001).

#### 2.3.4 Determination of antioxidant capacity

The 1,1‐diphenyl‐2‐picrylhydrazyl (DPPH·) scavenging activity of DPPE was evaluated according to Brand‐Williams et al. (1995). Briefly, 0.9 ml of the DPPH methanol working solution (6 10^−5^ M) was mixed with 0.6 ml of blank, sample, or standard (Trolox), kept in the dark for 30 min and the absorbance was recorded at 515 nm. The radical‐scavenging activity was expressed as millimole equivalent of Trolox /gram of freeze‐dried extract (mmol TE/g). The determination of ferric reducing antioxidant power (FRAP) was assessed as described by Benzie and Strain (1996). A FRAP solution containing 2.5 ml (10 mM) TPTZ [(2,4,6‐tri [2‐pyridyl]‐s‐triazine)] solution in 40 mM HCl, 25 ml of 0.3 M acetate buffer (pH = 3.6), and 25 ml FeCl_3_ (20 mM) was prepared. As much as 0.1 ml of DPPE was incubated at 37°C with 0.9 ml of FRAP solution for 30 min and then the absorbance was recorded at 593 nm. Results were expressed in micromole Trolox equivalents per gram (μmol TE/g) of freeze‐dried extract.

#### 2.3.5 Identification of phenolic compounds by LC‐MS

High‐performance liquid chromatography (HPLC) analyses were carried out using a Waters Alliance system (Waters Chromatography) coupled with a photodiode array detector (PDA) and interfaced with an electrospray ionization source (ESI) and a single quadrupole analyzer mass detector (MS). Sample separation (20 μl) was performed on an Acquity UPLC Kinetex® C18 (Phenomenex, Kinetex® 2.6 μm C_18_ 100 Å, 150 mm × 2.1 mm i.d.) at 25°C with a flow rate of 0.6 ml min^−1^. The injection volume was set at 1 μl. The mobile phase consisted of A: Water and B: Acetonitrile, both acidified with 0.05% formic acid. The following gradient was used: 0–5 min, 2% B; 5–10 min, 2%–15% B; 10–30 min, 15%–30% B, 30–38 min, 30%–98% B; 38–40 min, 98%–2% B; final isocratic step for 5 min at 2% B. PDA detection was performed in the 200–800 nm wavelength range, and the mass spectra were recorded in both negative and positive ion modes with the following setting: capillary voltage: 3 kV; cone voltage: 10 V, desolvation temperature: 500°C and ion source temperature: 150°C. The spectra were acquired in the m/z range of 80–1500 amu. Tentative identification of phenolic compounds was based on comparing ultraviolet (UV) absorption and mass fragmentation spectra with those of previously described compounds in *P. dactylifera* pits extracts (Ben Said et al., 2017; Farag et al., 2014; Otify et al., 2019).

### 2.4 Active alginate films' development, characterization, and stability

#### 2.4.1 Elaboration of alginate films containing DPPE

Alginate biopolymer was completely dissolved in distilled water at 70°C under mechanical stirring to obtain clear alginate film‐forming solutions at 3% (w/v). DPPE was added to film‐forming solutions previously cooled to 40°C to obtain the final concentrations of 0%, 10%, 20%, 30%, and 40% (w/w of alginate and DPPE). As much as 0.6 g of glycerol was added to alginate solutions under continuous agitation for 30 min. The concentrations of alginate, glycerol, and DPPE were selected based on preliminary assays where the processability, handling, and formation of homogeneous films were assured. Then, the obtained alginate‐based film‐forming solutions were degassed using an ultrasonic bath (BIOBASE). Twenty grams of each film‐forming solution was poured into 14 cm‐diameter Petri dishes and dried in a Venticell forced air convection oven (MMM group) at 40°C for 24 h to get films with an averaged thickness of 96,12 ± 0.88 μm. All films were kept in an environmental chamber at 50% RH (relative humidity) and 25°C for 1 week before testing.

#### 2.4.2 Fourier Transform Infrared analysis

The Fourier Transform Infrared (FTIR) spectra of alginate‐based films were recorded between 4000 and 500 cm^−1^ on an FTIR Bruker spectrometer (Equinox 55, Bruker Co.) with a diamond crystal Attenuated Total internal Reflectance (ATR) accessory. A total number of 32 scans were accumulated at 4 cm^−1^ resolution.

#### 2.4.3 Solubility

Film samples (2 cm × 2 cm) were dried at 105°C before being weighed (W_0_) and then soaked in 15 ml of deionized water for 24 h. All samples were delicately wiped and dried to a constant weight (W_1_) at 105°C. Water solubility was calculated as follows (Jouki et al., 2013):

$$\text{Solubility}\;\left(\%\right)=\frac{\left(W_{0}-W_{1}\right)}{W_{1}}\times 100\%$$

#### 2.4.4 Surface film wettability

Film contact angle was measured in quintuplicate to determine the film's surface wettability using a Pocket goniometer PGX (Sweden) following the sessile drop method (Gheribi et al., 2018). At least six measurements on each film surface were carried out at room temperature.

#### 2.4.5 Water vapor permeability

Water vapor permeability (WVP) measurements were performed according to the ASTM standard method E96/E96M (2015). Films with an exposed area of 26.42 cm^2^ were placed in permeation cells containing silica gel and stored in a controlled relative humidity (75% RH) and temperature (25°C) chamber. The WVP (g μm/m^2^ d kPa) was calculated as described by Gheribi et al. (2018). Three replicates were performed for each film formulation.

#### 2.4.6 Mechanical properties

Tensile strength (*TS*) and elongation at break (%*E*) were determined on rectangular film samples (15 mm wide × 100 mm long). Eight replicates of each film formulation were tested using an Instron 3345 universal testing machine (Massachusetts, USA) following the ASTM D882‐2 (2002). Measurements were performed at 23°C and 50% RH at a head speed of 20 mm min^−1^.

#### 2.4.7 Color

The color parameters, lightness (L*), red‐green (a*), yellow‐blue (b*), and the total color difference (ΔE) were determined at least five times for each film sample using a colorimeter CM‐5 (Konica Minolta). Color measurements were taken at four random points on each film.

#### 2.4.8 Light transmission and transparency

The light transmission of alginate‐based films was carried out by recording their ultraviolet–visible (UV–vis) spectra at the wavelength range between 200 and 800 nm. The transparency was calculated as follows:

$$\text{Transparency}=\frac{-\log\left(T600\right)}{X}$$

where *T600* is the transmittance at 600 nm and *X* is the film thickness (mm).

#### 2.4.9 Morphological characterization

The morphological analysis of the surface and cross‐sections of alginate‐based films was performed using a FEI Quanta 200 scanning electron microscope (FEI Company). Film samples were gold‐coated using “Sputter Coater S150” under vacuum and examined using an accelerating voltage of 20 kV.

#### 2.4.10 Film stability

Total phenolic content (TPC), as well as antioxidant activity (DPPH and FRAP assays) were determined for films enriched with DPPE on days 30, 60, and 90 to test the stability of the films.

### 2.5 Statistical analysis

Data were subjected to one‐way analysis of variance (ANOVA) using SYSTAT (Systat software Inc.). Means comparisons were performed through 95% Fisher's least‐square difference (LSD) intervals.

## 3 RESULTS AND DISCUSSION

### 3.1 Characterization of the DPPE extract

#### 3.1.1 Phenolic, condensed tannin, and anthocyanin contents

Total phenolic (TPC), condensed tannin (CTC), and anthocyanin (TAC) contents as well as the DPPH scavenging activity and ferric reducing power are summarized in Table 1. The TPC was 33.1 mg eq. GAE/g extract. The TAC was found to be 0.67 mg CGE/g extract, whereas CTC evaluated by Stiasny's index was 27.9%. Phenolic content and antioxidant capacities of date pit extracts are highly dependent on many factors including *P. dactylifera* origin, varieties, developmental stage, and extraction techniques (Echegaray et al., 2021). Our results agree with the findings of Mostafa et al. (2022) who reported a TPC value of 38.18 ± 0.86 mg GAE/g DW for date seeds *var*. Khalas from the United Arab Emirates. In the work of Bijami et al. (2020), changes in phenolic and tannin contents of date seeds *cv*. Mozafati were investigated. The results showed that TPC and CTC increased remarkably during the developmental stages, reaching the highest values of 27.38 mg/g DW and 17.7 mg/g DW, respectively.

**TABLE 1:** Date palm pit extract (DPPE) characterization of phenolic contents and antioxidant activity

Parameters	Units	Values
Total phenolic content (TPC)	mg GAE. g^−1^ ^a^	33.14 ± 1.04
Condensed tannin content (CTC) (Stiasny's index, SI)	%	27.89 ± 0.71
Total anthocyanin content (TAC)	mg CE. g^−1^	0.67 ± 0.04
DPPH^•^ radical‐scavenging capacity	mmol TE. g^−1^	2.52 ± 0.19
Ferric reducing antioxidant power (FRAP)	μmol TE. g^−1^	375.85 ± 10.17

*Note*: Values are given as mean ± standard deviation (*n* = 3).

^a^ Values expressed on a freeze‐dried extract weight basis.

#### 3.1.2 LC–MS analysis

**FIGURE 1:**
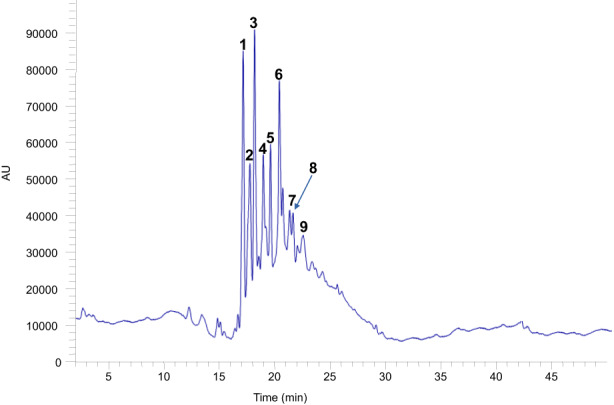
LC‐PDA‐TIC (liquid chromatography–photodiode array detector–total ion chromatogram) profile of the date palm pit extract (DPPE). Identification of numbered peaks is given in Table 2.

The qualitative analysis of the phytochemicals in DPPE was performed by using an LC–MS system equipped with an ESI source and a single quadrupole analyzer. Positive and negative modes were used for MS experiments. The UV chromatogram of DPPE is shown in Figure 1. Retention time and mass spectral data of the detected phenolic compounds are presented in Table 2.

**TABLE 2:** Identification of the main phenolic compounds in date palm pit extract (DPPE) (retention times, mass spectrometry (MS) spectral fragments, and example structures)

N°	*Rt* (min)	[M − H]^−^/[M + H]^+^ (m/z)	Fragments (m/z)	Chemical formula/Molecular weight (Da)	Tentative identification
1	17.9	577 [M − H]^−^/579 [M + H]^+^	289 [M − H‐288]^−^ 425 [M − H‐152]^−^	C_30_H_26_O_12_ (578)	Procyanidin dimers 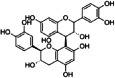
5	19.5				
2	17.9	865 [M − H]^−^/867 [M + H]^+^		C_45_H_38_O_18_ (866)	Procyanidin trimers 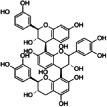
3	18.1		577[M − H‐288]^−^		
4	19.1		713 [M − H‐152]^−^		
6	20.2		695 [M − H‐152‐18]^−^		
8	21.9		579 [M + H‐288]^+^		
9	22.5				
7	20.8	335 [M − H]^−^	179 [M − H‐156]^−^ 161 [M − H‐174]^−^	C_16_H_16_O_8_(336)	O‐caffeoylshikimate 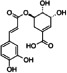

Peaks **1** (*Rt* at 17.1 min) and **5** (*Rt* at 19.5 min) showed a λ_max_ at 280 nm and [M–H]^−^ molecular ions at m/z 577. MS spectrum showed a prominent fragment at m/z 577 corresponding to [M–H‐288]^−^ interpreted as a loss of an (epi)catechin molecule, a second less intense at m/z 425, which indicates a loss of 152 amu generated by retro‐Diels–Alder fission of a flavan‐3‐ol C‐ring (de la Cádiz‐Gurrea et al., 2014). Both peaks presented an ion fragment at m/z 289 corresponding to a deprotonated (epi)catechin. Therefore, **1** and **5** were assigned, respectively, to dimer procyanidins (Ben Said et al., 2017; Escobar‐Avello et al., 2019).

Accordingly, peaks **2** (*Rt* at 17.9 min), **3** (*Rt* at 18.1 min), **4** (*Rt* at 19.1 min), **6** (*Rt* at 20.2 min), **8** (*Rt* at 21.9 min), and **9** (*Rt* at 22.5 min) were assigned to trimer procyanidins. They shared the same UV absorption maxima at 280 nm and molecular ions [M − H]^−^ and [M + H]^+^ at m/z 865 and 867, respectively. They also showed the typical presence of [M–H‐288]^−^ at m/z 577 and [M–H‐288‐288]^−^ at m/z 289 which corresponds to the loss of two (epi)catechin molecules.

Peak **7** (*Rt* at 20.8 min) with UV absorption maxima at 280 and 324 nm produced molecular ions [M − H]^−^ at m/z 335. In the negative ionization mode, the presence of two fragment ions at m/z 179 and 16 indicates neutral losses of 156 Da (shikimic acid–H_2_O) and 174 Da (shikimic acid). Based on this information, compound **7** was tentatively identified as O‐caffeoylshikimic acid (Ben Said et al., 2017; Farag et al., 2014).

### 3.2 Characterization of alginate films containing DPPE

#### 3.2.1 FTIR analysis

**FIGURE 2:**
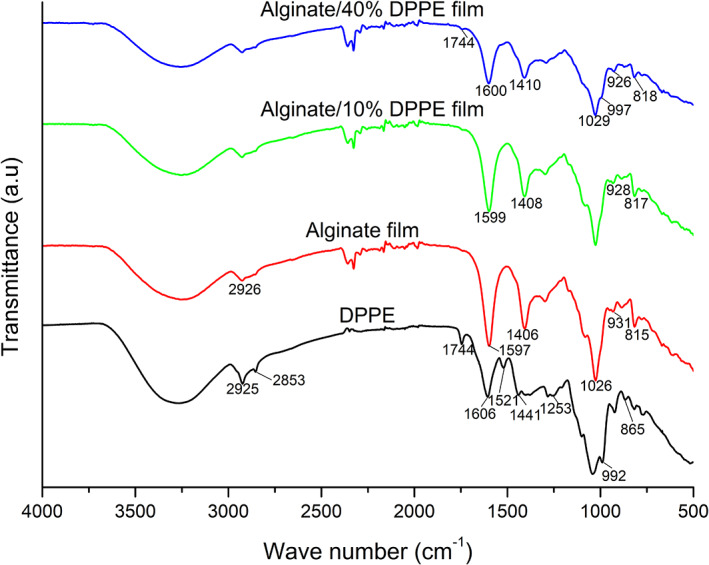
Attenuated total internal reflectance–Fourier transform infrared (ATR–FTIR) spectra of the date palm pit extract (DPPE), neat alginate film (control), and alginate loaded with 10% and 40% DPPE.

The FTIR spectrum of DPPE showed a broad band at 3280 cm^−1^ relative to the –OH groups stretching and two peaks at 2925 and 2853 cm^−1^ related to asymmetric and symmetric stretching vibrations of C–H, respectively (Figure 2). A peak assigned to C=O groups of hemicellulose and lignin appeared at 1477 cm^−1^ (Belala et al., 2011). The absorption bands at 1606, 1521, and 1441 cm^−1^ may correspond to aromatic ring skeleton stretching and vibration (Zhuang et al., 2020). The band at 1253 cm^−1^ may be ascribed to O–C–H, C–C–H, and C–O–H bending (El‐Hendawy, 2006). The peaks between 1200 and 950 cm^−1^ are assigned to C–O stretching vibration. C–H vibration of cellulose/hemicellulose is presented at 865 cm^−1^.

The FTIR spectrum of alginate film is consistent with alginate FTIR spectra reported in the literature (Augusto et al., 2018; Luo et al., 2019), and displayed characteristic bands at 3253, 2926, 1597, 1406, 1026, and 815 cm^−1^ corresponding to –OH groups, C–H stretching, asymmetric COO– stretching, symmetric COO– stretching, C–O–C stretching, and mannuronic acid residues, respectively.

**FIGURE 3:**
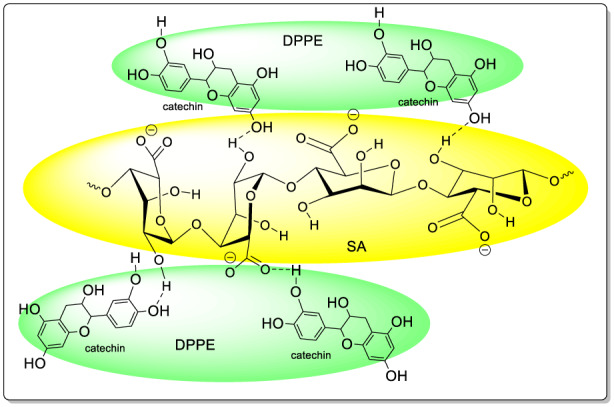
Schematic illustration of the interaction between sodium alginate (SA) and date palm pit extract (DPPE).

The changes induced by incorporating increasing amounts of DPPE in alginate films are shown in Figure 2. A noticeable decrease in the intensity of alginate characteristic bands was observed upon DPPE incorporation. The intensity of peaks ascribed to asymmetric and symmetric –COO stretching and C–O–C stretching decreased with increasing DPPE loading and shifted to higher frequencies (1600, 1410, and 1029 cm^−1^, respectively). On the other hand, the appearance of a peak at 1744 cm^−1^, relative to C=O groups of hemicellulose and lignin from DPPE, in alginate/DPPE films confirmed the presence of DPPE in composite films. All these changes implied the interaction between the alginate matrix and DPPE through hydrogen bonds (Figure 3). The hydrogen bonds involve hydroxyl groups of polyphenols (i.e., catechin) and both hydroxyl and COO‐ groups of alginate (Plazinski & Plazinska, 2011).

#### 3.2.2 SEM analysis

**FIGURE 4:**
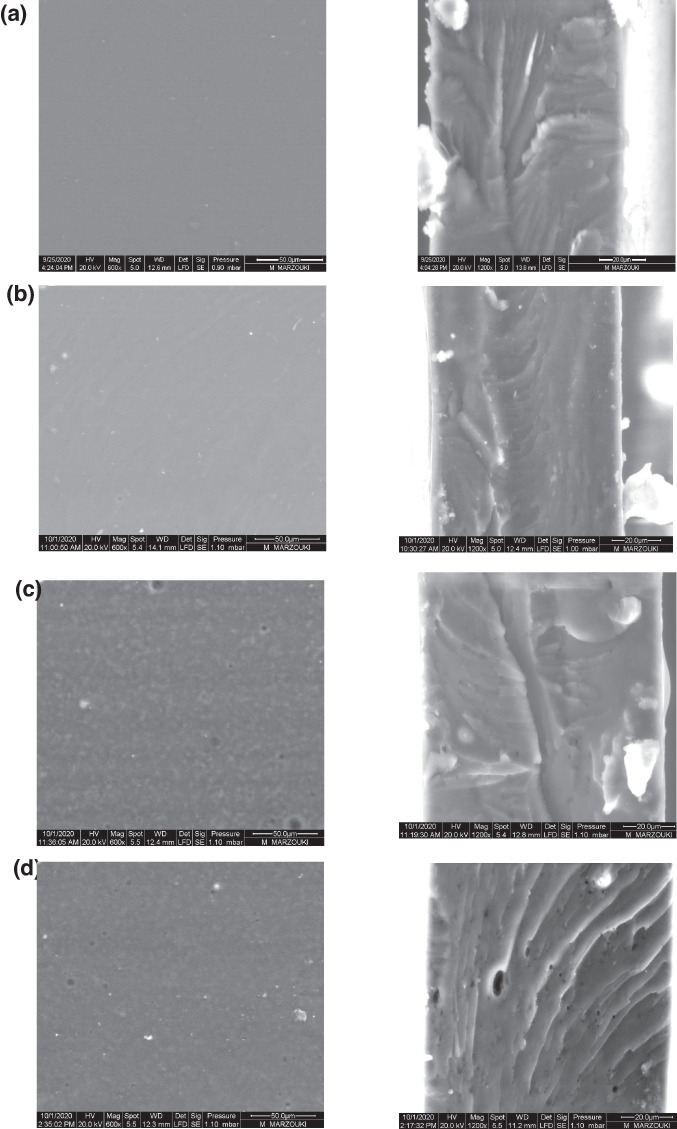
Scanning electron microscopy (SEM) micrographs of surface (×600) and cross‐section (×1200) of (a) neat alginate film, (b) alginate/10% date palm pit extract (DPPE) film, (c) alginate/30% DPPE film, and (d) alginate/40% DPPE film.

Scanning electronic microscopy (SEM) micrographs of the surface and cross‐sections of alginate films incorporating different DPPE concentrations are shown in Figure 4. Neat alginate films had a glazed and homogeneous surface. Likewise, alginate films incorporating 10% DPPE exhibited a compact cross‐section and a relatively homogeneous surface. However, increasing DPPE loading resulted in an increase in surface discontinuities and heterogeneities mainly due to the agglomeration of DPPE particles. Moreover, the cross‐section of films incorporating high DPPE loading displayed several pinholes and bulges which may explain the obtained low mechanical strength and high solubility, WVP and surface wettability of films containing a high DPPE content (>20%). These observations were consistent with those of (Ruan et al., 2019) who found that increasing epigallocatechin gallate loading led to an increase in surface heterogeneities and roughness of alginate films due to alginate–catechins' coalescence. Conversely, Luo et al. (2019) noticed a more compact and dense structure after incorporation in the alginate matrix of increasing amounts of ethanolic guava leaf extract.

#### 3.2.3 Solubility

Film solubility is intimately related to water barrier packaging performance and dictates its biodegradability behavior. Neat sodium alginate film displayed the highest water solubility (*p* < .05) and such high alginate hydrophilic character in high humid environments has been highlighted in the literature (Abdollahi et al., 2013; Costa et al., 2018). The incorporation of DPPE in the alginate films significantly decreased solubility values by 63% and 37% at 10% and 40% DPPE, respectively, compared with the neat alginate film (*p* < .05). However, no significant differences were observed between the solubility of alginate films incorporating 30% and 40% DPPE (Table 3). The improvement in water barrier properties of alginate films with DPPE incorporation is indicative of the formation of a cross‐linked film structure due to intermolecular bonds between alginate chains and DPPE as confirmed by the ATR–FTIR analysis (Figure 2). A similar trend has been observed by Augusto et al. (2018) who explained the decrease in the solubility of alginate films incorporating 0.5% seaweed extract (SE) by the establishment of hydrophobic interactions between constituents of SE and alginate chains. Conversely, the incorporation of guava leaf extracts (GLE) led to an enhancement in the solubility of sodium alginate films due to the hydrophilic character of polyphenols in GLE (Luo et al., 2019).

**TABLE 3:** Physical and mechanical properties of alginate films with different DPPE loading

Films	Solubility (%)	Contact angle (°)	WVP (g.μm/m^2^.d. kPa)	TS (MPa)	% E
Neat sodium alginate	86.98 ± 1.10^a^	33.06 ± 1.10^c^	56.62 ± 4.14^b^	30.93 ± 3.42^b^	13.86 ± 1.18^e^
Alginate–10% DPPE	32.46 ± 0.78^c^	67.05 ± 6.00^a^	44.00 ± 1.41^a^	46.37 ± 4.02^a^	20.22 ± 0.71^d^
Alginate–20% DPPE	31.43 ± 0.98^c^	69.77 ± 4.00^a^	37.63 ± 2.92^a^	41.64 ± 1.06^a^	24.44 ± 2.20^c^
Alginate–30% DPPE	53.07 ± 0.74^b^	59.75 ± 5.00^b^	55.20 ± 2.69^b^	29.92 ± 6.69^bc^	28.71 ± 2.90^b^
Alginate–40% DPPE	54.60 ± 0.61^b^	57.02 ± 4.50^b^	58.01 ± 2.80^b^	25.46 ± 0.81^c^	37.24 ± 2.29^a^

*Note*: All values are presented as mean ± standard deviation; Different lowercase letters in the same column indicate significant differences (*p* < .05).

Abbreviation: DPPE, date palm pit extract.

#### 3.2.4 Water contact angle

Water contact angle indicates the film's surface hydrophilicity. Neat sodium alginate film exhibited the lowest contact angle (~33°) and thus the highest hydrophilicity. DPPE incorporation significantly increased water contact angle (*p* < .05). The water contact angle of alginate films incorporating 10%–20% DPPE was 2 times higher than that of neat alginate film (Table 3). However, increasing DPPE content beyond 20% decreased water contact angle but this latter remained 1.7 times higher than that of alginate film. The observed increase in the water contact angle and subsequently decrease in surface wettability may be attributed to the interaction between alginate polymer and DPPE, which may reduce the number of available polar groups on the film surface and consequently decrease the overall hydrophilic character of the alginate film. Likewise, Aloui et al. (2019) and Manrich et al. (2017) reported a significant increase in the water contact angle of pectin and caseinate films with increasing the lipid fraction of tomato pomace and tomato cutin, respectively. However, our results disagree with the findings of Augusto et al. (2018) who noticed an increase in alginate and chitosan films' wettability with the incorporation of 0.5% SE due to the polar compounds present in the extract.

#### 3.2.5 Water vapor permeability

Water vapor permeability reflects the capacity of a packaging film to inhibit moisture transfer. The incorporation of DPPE in the alginate films significantly decreased WVP values by 23% and 34% at 10% and 20% DPPE, respectively, compared with the neat alginate film. The improvement in water vapor resistance properties of alginate films with DPPE incorporation (10%–20%) is indicative of the formation of a dense and compact film network due to interactions between alginate chains and DPPE and allows their use in many food packaging applications. According to Babapour et al. (2022), the improved moisture barrier properties of potato starch films due to the incorporation of fennel essential oil and zinc oxide nanoparticles may explain their ability to reduce moisture loss of pistachios and the decomposition of carbohydrates and fats in kernel tissues.

However, no significant differences (*p* > .05) were observed between the WVP of neat alginate films and those incorporating 30% and 40% DPPE (Table 3). Increasing DPPE content beyond 20% induced structural changes in the polymeric matrix, promoting the diffusion of water vapor through pinholes and bulges as revealed by SEM micrographs (Figure 4).

#### 3.2.6 Mechanical properties

Mechanical properties of packaging material, viz., mechanical strength and flexibility are correlated to its ability to resist external stresses and cracks and preserve packaging's physical and functional performances during transport and food storage. TS and %E results are shown as a function of DPPE content (Table 3). DPPE incorporation at 10% and 20% significantly increased TS of alginate films by 50% and 35%, respectively. However, increasing DPPE content beyond 30% decreased TS by 18% compared with the neat alginate film. The increased mechanical strength of alginate films incorporating DPPE (10%–20%) may be attributed to the formation of a strong and cohesive cross‐linked structure resulting from the interactions between alginate chains and extract components through hydrogen bonding and hydrophobic interactions (Figure 3). TS results agree with those of solubility and water contact angle as high DPPE amounts (beyond 20%) increased film solubility and surface wettability. High DPPE content (>20%) may induce surface discontinuities and heterogeneities in the polymeric matrix resulting from the agglomeration of DPPE particles, as revealed by SEM micrographs (Figure 4), leading to weaker films. Similarly, Biao et al. (2019) noticed that the mechanical strength of alginate films increased after the addition of low amounts of tea polyphenols (<2% w/w) but decreased at high amounts of polyphenols. On the other hand, DPPE incorporation into alginate films led to an increase in their flexibility (Table 3). A similar trend was observed for chitosan films after the incorporation of 0.5% (w/v) *Codium tomentosum* extract which by acting as a plasticizer increased chain mobility and film extensibility (Augusto et al., 2018). An increase in %E of starch films due to the addition of fennel essential oil was also observed by Babapour et al. (2021) and explained by its softening effect, interfering with potato starch interactions.

#### 3.2.7 Optical properties

The optical properties of packaging films greatly affect product appearance and thus consumer acceptance. The optical properties of alginate and alginate/DPPE are listed in Table 4. DPPE incorporation into alginate films decreased their lightness and this decrease in L values was more pronounced in films containing higher DPPE amounts (*p* < .05), but increased a* and b* values (*p* < .05). As the DPPE content increased, the alginate films become darker, redder (+a*), and yellower (+b*). This may be related to the characteristic brown color of DPPE, which increased ΔE values of alginate films (*p* < .05), being linearly correlated to DPPE content (R^2^ = 0.9676). Our results agree with the findings of Luo et al. (2019) who reported an increase in a*, b*, and ΔE values with increasing aqueous and ethanolic guava leaf extract concentration from 10% to 20% in alginate films.

**TABLE 4:** Optical properties and physical appearance of alginate films with different DPPE loading

Films	L*	a*	b*	ΔE	Transparency	Photographs
Neat sodium alginate	95.77 ± 0.84^a^	−0.29 ± 0.06^d^	4.00 ± 1.35^d^	1.27 ± 0.14^c^	0.58 ± 0.04^c^	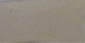
Alginate–10% DPPE	88.78 ± 1.57^b^	3.23 ± 0.64^c^	9.31 ± 1.01^c^	3.06 ± 0.43^b^	1.31 ± 0.18^b^	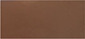
Alginate–20% DPPE	83.87 ± 3.61^c^	4.75 ± 0.71^b^	18.41 ± 1.55^b^	3.55 ± 0.41^b^	1.43 ± 0.20^b^	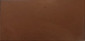
Alginate–30% DPPE	83.56 ± 2.07^c^	7.10 ± 0.73^a^	19.95 ± 1.72^b^	5.82 ± 0.43^a^	1.74 ± 0.22^a^	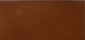
Alginate–40% DPPE	82.81 ± 1.48^c^	7.68 ± 0.50^a^	24.63 ± 2.18^a^	6.57 ± 0.88^a^	1.74 ± 0.15^a^	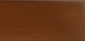

*Note*: All values are presented as mean ± standard deviation; Different lowercase letters in the same column indicate significant differences (*p* < .05).

Abbreviation: DPPE, date palm pit extract.

DPPE content increased the transparency values of alginate films (*p* < .05), which means that composite films based on alginate/DPPE had lower transparency and higher opacity than neat alginate films (Table 4). The good light barrier properties of alginate/DPPE films may be exploited for the protection of foods susceptible to photooxidation. These results are consistent with those of Augusto et al. (2018) and Biao et al. (2019) who noticed an improvement in light barrier properties of alginate films after the incorporation of *C. tomentosum* extract (0.5% w/v) and tea polyphenols (1%–5% w/w), respectively.

### 3.3 Antioxidant capacity and stability of Alginate–DPPE films

**FIGURE 5:**
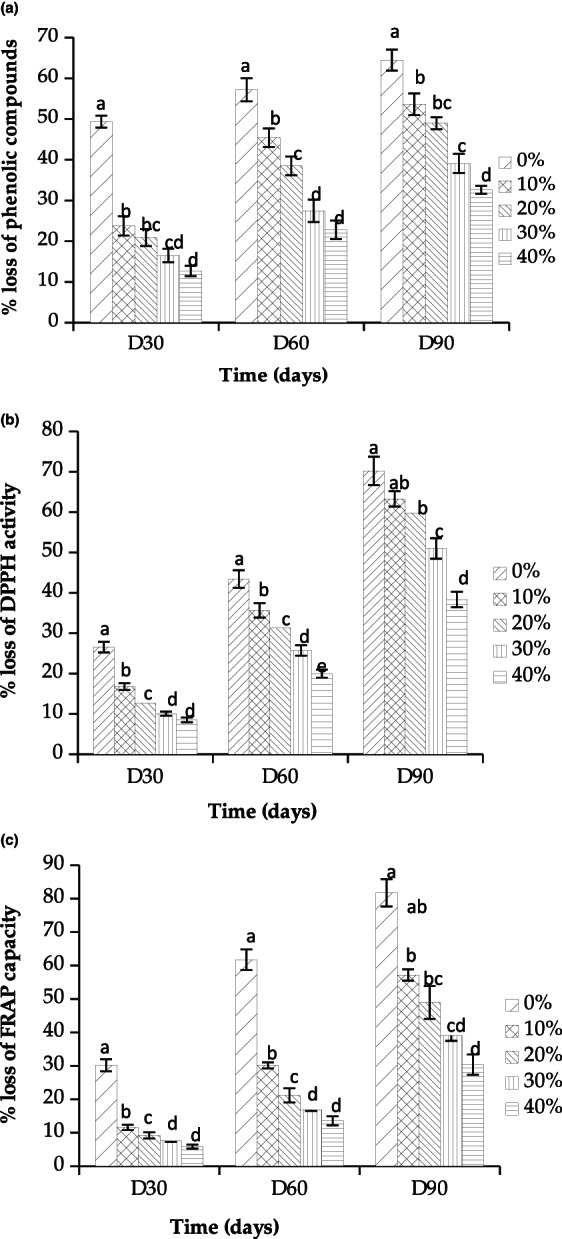
Percentage of loss of phenolic compounds (a), 1,1‐diphenyl‐2‐picrylhydrazyl (DPPH) activity (b), and ferric reducing antioxidant power (FRAP) capacity (c) during storage for 90 days for alginate films incorporating date palm pit extract (DPPE). Values are means of 3 replicates ± standard deviation (SD). Values with different letters are significantly different (*p* < .05).

Figure 5 presents the percentage of phenolic compounds and antioxidant capacity loss (DPPH and FRAP assays) for the films enriched with 10%, 20%, 30%, and 40% DPPE on days 30, 60, and 90. Films without DPPE were used as control.

The control film presents the highest loss of phenolic content with 49, 57, and 64 on days 30, 60, and 90, respectively. The film containing 40% DPPE showed the lowest loss of phenolic content (13% on day 30 and 33% on day 90), whereas films containing the lowest percentage of DPPE showed the highest loss (24% and 54% on days 30 and 90, respectively). The same trend was observed for DPPH and FRAP assays. The loss of DPPH scavenging activity was 9% and 38% on days 30 and 90, respectively, for the film containing 40% DPPE, and reached 27% and 70% for free DPPE film. For the ferric reducing antioxidant power, the highest loss of FRAP values was observed for free DPPE films and the lowest one was observed for 40% DPPE films. In summary, we noted that as the concentration of DPPE in the film increases, the loss of TPC and antioxidant activities (DPPH and FRAP) decrease.

These results show that polyphenols from DPPE added to alginate films may act as antioxidants enhancing the packaging effectiveness in a concentration‐dependent manner (Rhimi et al., 2018). Previous work showed a close relationship between TPC and the antioxidant activity of date pits extracts (Alqahtani et al., 2021). Eça et al. (2015) have studied phytochemical contents and antioxidant capacity for 3 months' shelf‐life storage of pectin films containing different fruit extracts. They reported a significant decrease in phenolic compound concentrations and subsequently a decrease in the antioxidant capacity of pectin films at the end of the monitoring period. Previous stability studies of ascorbic acid supplemented in alginate films showed that the degree of hydrolysis of this antioxidant is dependent on the glycerol level as well as proportions of flexible blocks within the polymer structure (De'Nobili et al., 2013).

In our study, catechin derivatives are the main phenolic constituents of DPPE. They are found in many plants and are known to act as strong antioxidants (Ruan et al., 2019). A packaging film based on polyvinyl alcohol (PVA)–starch impregnated with catechins from green tea was studied by Wu et al. (2010). They found that the inclusion of catechins induces significant retardation in oxidation as revealed by a decrease in thiobarbituric acid reactive substances (TBARS). Moreover, catechins induced inhibition of the growth of airborne microorganisms. Roedig‐Penman and Gordon (1997) reported a similar antioxidant activity during 40 days of storage of oil when they compared the effect of aqueous extract of tea and butylated hydroxytoluene (BHT), a synthetic antioxidant standard. Because of their high molecular weight and the fact that they are nonvolatile compounds, catechins incorporated in packaging films can diffuse between these active packaging materials and the food matrix and/or partition at the interface (Ruan et al., 2019).

## 4 CONCLUSIONS

Active films were prepared by incorporating DPPE from date pits into an alginate matrix. The effects of DPPE loading on the physicochemical, mechanical, and antioxidant properties of alginate films and their stability during storage were studied. The incorporation of 10% DPPE in the alginate films significantly decreased solubility values by 64%, enhanced water contact angle values and mechanical strength by 103% and 50%, respectively, and exhibited good light barrier properties. The improvement in water vapor barrier properties and mechanical strength of alginate films incorporating DPPE (10%–20%) may be attributed to the formation of a strong and cohesive cross‐linked structure resulting from the interactions between alginate chains and extract components through hydrogen bonding and hydrophobic interactions as indicated by FTIR and SEM analyses. However, alginate films containing a high DPPE content (>20%) displayed lower water vapor barrier properties and mechanical strength as well as higher solubility and surface wettability. Increasing DPPE loading led to a decrease in the loss of in vitro antioxidant activity. Catechin derivatives, the main phenolic constituents of DPPE, can act as strong and natural antioxidants when incorporated into packaging films by retarding lipid oxidation in foods during storage. Overall, alginate films incorporated with DPPE (10%–20%) exhibited the highest physical and mechanical properties and good antioxidant and light barrier properties, hence they may be successfully used to improve the quality of foods high in unsaturated fat such as nuts and seeds.

## FUNDING INFORMATION

This research was funded by the VallCET project (2021–2023), which is funded through the PRIMA (Partnership for Research and Innovation in the Mediterranean Area) by the Ministry of Higher Education and Scientific Research (MHESR, Tunisia).

## CONFLICT OF INTEREST

The authors declare no conflict of interest. The funders had no role in the design of the study; in the collection, analyses, or interpretation of data; in the writing of the manuscript, or in the decision to publish the results.

## Data Availability

Data available on request from the authors.
